# Comprehensive proposal for a pedestrian-focused road safety inspection

**DOI:** 10.1016/j.heliyon.2025.e42021

**Published:** 2025-01-21

**Authors:** Melva Guadalupe Herrera Godina, Guillermo González Estevez, Gilberto Silva Bañuelos, Luis Eduardo del Moral Trinidad

**Affiliations:** a Public Health, University Center for Health Sciences (CUCS), University of Guadalajara, Guadalajara, Mexico; b Social Sciences, University Center for Health Sciences (CUCS), University of Guadalajara, Guadalajara, Mexico; c Public Health Sciences, University Center for Health Sciences (CUCS) University of Guadalajara, Guadalajara, Jalisco, Mexico

## 1 Introduction

Traffic events in which pedestrians are injured by motor vehicle collisions have been analyzed from different perspectives, highlighting the constant search for risk factors that influence the frequency of traffic crashes, focusing on the characteristics of the individual or the vehicle. However, there are other elements external to the characteristics of the pedestrian, motorist and vehicle that influence traffic crashes, such as the characteristics of the environment where the event took place [1,2]. Road safety inspections (RSI) serve this purpose by systematically evaluating these external elements to identify hazards and potential improvements for preventing accidents [3].

A road safety inspection is a tool used to evaluate existing roads and focuses on finding safety deficiencies that may have developed over time. These tools are necessary to perform ongoing evaluations of the roads, as traffic and pedestrian patterns can vary from one time to another [4]. This methodology often involves the use of checklists to assess different features and roads conditions [5]. Road safety inspections can incorporate a wide range of elements such as road geometry, traffic volume, roadside features, signage, and pedestrian facilities. Identifying these elements becomes critical to ensure safe travel for both motor vehicle users and pedestrians. By thoroughly assessing these elements, road safety inspectors can identify potential hazards and develop target interventions to improve road safety for all users [6].

To fulfill its purpose, road safety inspections need to be tailored to the context in which they are conducted. For instance, in rural areas, RSIs may focus on elements related to wildlife crossings, unpaved road conditions, and limited visibility due to insufficient public lighting [5]. Conversely, urban RSIs should emphasize the evaluation of multimodal interactions between cyclists, pedestrians, and various motor vehicle users such as cars and motorcycles [7].

These evaluation tools, as well as others available, should be aligned with regional safety policies to ensure an integrated evaluation that considers local conditions, requirements, and needs [8]. Members of the United Nations Economic Commission for Europe (UNECE) have incorporated road safety policies into their national laws, highlighting the need to constantly evaluate roads and ensure they adequately function for all users. By incorporating these policies into national legislation, countries within the UNECE region have demonstrated a commitment to prioritizing the safety of all road users, including pedestrians, cyclists, and motorists [9].

In Mexico, according to recommendations from the Secretariat of Communications and Transport, these road safety inspection tools should include an assessment of elements such as intersections, acceleration and deceleration ramps, lighting and signage, spaces for pedestrians and cyclists, rest areas, public transportation facilities, among others [10]. However, the existing road safety inspection approach primarily focuses on the physical road structure and related elements but fails to comprehensively address all the factors that could pose hazards for pedestrians, who are the most vulnerable road users and require greater consideration [11].

Including pedestrians in road safety evaluations is key to generate adequate data to develop tailor traffic safety policies in our context. Evidence shows that vulnerable users have experience higher rates of injuries and fatalities in urban areas comparing to drivers, especially at intersections with high vehicular flow [12,13]. Moreover, pedestrian focused interventions like *Pasos seguros* program in Mexico shown that street-level design adjustments, including sidewalk widening and traffic light optimization, can reduce accidents that involve pedestrians’ users [13].

Mexico's current road safety inspections often lack a focus on pedestrians' specific hazards, focusing mainly on vehicle occupant safety [14]. Since pedestrians’ injuries represent a significant portion of roads traffic deaths in our context there is a need to develop tailored tools that allow to evaluate existing roads on a pedestrian center design [14,15] Thus, the objective of this article was to design a road safety inspection questionnaire that analyzes the elements present on roads to identify opportunities for improving road safety.

## 2 Methodology

The road safety inspection was developed in Guadalajara, Jalisco, Mexico. The methodology drew upon existing tools used to diagnose safety issues related to road design elements. This included approaches proposed by Fundación MAPFRE and the Mayor's Office of Bogotá, D.C., which identify different types of evaluations depending on the road site phase, such as planning, project, construction, opening, and operation [16,17]. Since the focus of this study was on developing an RSI, all the sites analyzed were in the operation phase, and the data collection questionnaire was designed accordingly.

As a first approach, we conducted a literature review to identify the most relevant elements that other studies have considered as risk factors for pedestrians. This allowed the incorporation of a more comprehensive set of factors that could influence the occurrence of pedestrian crashes. Specifically, a systematic review was undertaken to identify built-environment risk factors associated with pedestrian-motor vehicle collisions. Subsequently, additional risk factors were identified using the snowball method from the references cited in the articles obtained during the review. The selection of variables for inclusion in the pedestrian road safety inspection tool was guided by the urban network theory, focusing on elements that either enhance or hinder the continuity of pedestrian mobility [18].

Secondly, to create our collection questionnaire, it was necessary to have a uniform definition of each of the variables used. This was done to ensure consistency and clarity throughout the data collection process. Thus, Annex II includes the definitions of these variables, providing a clear and standardized framework to avoid any ambiguities or discrepancies in how the different elements were consider and analyzed.

After unifying the variables and their definitions, the initial draft of the data collection questionnaire was designed. This initial draft led to the development of a data collection form comprising 7 sections, which were organized based on the elements and geometric characteristics of the road, as well as an analysis of the infrastructure. The sections included are: 1) Geometric design features of the roadway, 2) Signage, 3) Pedestrian subsystem features, 4) Traffic circle characteristics, 5) Bus stop characteristics, 6) Traffic light characteristics, and 7) Speed reducer features.

Then we proceed to evaluate the draft questionnaire in a pilot study that took place in four intersections that were randomly selected within the Guadalajara Metropolitan Zone. After testing on each zone, the feedback from the pilot study enabled us to refine the questionnaire, incorporate any element that were missing, and clarify definitions or instructions that were unclear to the evaluators.

The testing and evaluation phase of the questionnaire took place in two stages. In the first stage, 4 intersections were randomly selected within the Guadalajara Metropolitan Zone and the information was collected.

At the first intersection, located at Capuchina and Prolongation Gigantes in Tonalá, the research team met challenges in deciding the road type. Without a detailed map of the metropolitan area, it was difficult to assess the connectivity of this specific road. Additionally, the cobblestone surface of the road, which had not been initially considered, led to several complications. The inability to decide the number of lanes or their widths resulted in the elimination of these variables and their replacement with the overall street width, as well as the addition of the road surface type as a new variable. The lack of lane demarcation also posed a problem, as it made it impossible to calculate the turning radius on two-way streets. The intersection also highlighted the need to consider the continuity of the sidewalks, as the team initially only asked about the presence of slopes, steps, and obstructions, but did not account for the fact that some streets have continuous sidewalks while others have interruptions.

The second sampling point is the intersection of Fray Junipero Sierra and Alfonso Reyes streets, in the municipality of Guadalajara. At this point, the main problem faced was the abundant vehicular traffic, which made it impossible to measure the width of the street, especially because of the instrument used (the flexometer), which made it necessary to look for another measuring instrument, and a digital distance meter was chosen.

The third site analyzed was the intersection of Boulevard Marcelino García Barragán and Río Tajo, in the municipality of Tlaquepaque. At this point, it was reconsidered to substitute the height variable of the vertical signs and the continuity variable of the horizontal signs, by visibility in the case of the vertical signs, and by state of preservation of the horizontal signs.

The intersection located at Angulo and Pedro Loza, municipality of Guadalajara, was the fourth point to be consulted, from its observation it was highlighted the lack of consideration of the road surface conditions, that is, if there are potholes in the place, a variable that was included in the final format of the collection form.

The field observation included the variable of the contraflow lane, which is not considered in the methodologies for the construction of road safety audits taken as a reference. In relation to the bus stop, the need to remain at the site for a minimum of 15 min due to the transit of public transport lines became palpable, since at some points and although the bus stop is not signaled, the intersection can act as a bus stop by habit, and that if this variable is overlooked, it would be underestimated.

When testing and evaluating the form in the field, it was necessary to modify the form and substance of the form. The modified format includes 45 elements of the road system distributed in 7 sections (Annex III). The data collection form consisted of the following sections:

1. Section of the geometric design characteristics of the roadway
2. Section on the characteristics of the traffic circle.
3. Signaling section
4. Section of the bus stop characteristics
5. Section of the characteristics of the traffic lights.
6. Section of speed reducers
7. Section on pedestrian subsystem characteristics

## 3 Discussion

The aim of this article was to design a road audit report based on the various elements that constitute pedestrian safety, and which is reinforced through a methodology that allowed the integration of these elements into a single report.

The development of the work was based on the methodologies proposed by Fundación MAPFRE and the Mayor's Office of Bogotá, D.C. (2005), which have been used in other works on road audits [16,19], finding a solid construction for the evaluation of road safety from the pedestrian's perspective; where the design stages, the construction phase and the operation phase are included. In this way, the elements included are articulated to the urban reality of our context, considering the particularities that drivers and pedestrians experience in their transit through the city and that, in the end, allows the identification of road risks for both.

Due to the complexity of the roadways in our context, the proposed work offers the opportunity to be applied individually to each of the streets to be studied. This allows for a more detailed and comprehensive evaluation, as the instrument can be applied to each of the streets that make up an intersection. This detailed approach enables a deeper understanding of the safety conditions and risk factors present along the different components of an intersection, rather than just assessing the intersection. By analyzing each street separately, the assessment can identify specific issues and tailor mitigation strategies to the unique characteristics of that roadway segment.

Also, in line with other road audit evaluations [17,20], the sections of this report are based on the factors identified after a targeted literature review. It should be noted that the intention of the authors was to go beyond just the interest of the roadway, but also to recognize the active role of the pedestrian to present a work that articulates the factors of both vehicles and pedestrians on the various roadways.

In contrast to other road evaluations that focus solely on the assessment of road infrastructure, this work proposes a comprehensive approach that incorporates the role of elements that could pose a risk for pedestrians. Specifically, this RSI goes beyond just evaluating the physical road conditions and infrastructure, and considers factors related to pedestrian safety and mobility. By including elements such as the characteristics of traffic signals, bus stops, and speed reducers, as well as the conditions of the pedestrian subsystem, this work provides a more holistic assessment of the road environment and the potential risks faced by pedestrians [21]. This approach aims to identify a broader range of safety issues and allow for the development of more targeted mitigation strategies to improve the overall road safety experience for all users.

The application of this road audit report is expected to contribute significantly to the identification of a wide range of risk factors that threaten the safety of pedestrians in mexican urban areas and alike regions. Consequently, the insights gained from this assessment can inform the development and implementation of targeted mitigation measures, aimed at improving the overall road safety conditions and enhancing the pedestrian experience in urban environments.

On the other hand, we recognize the diversity that exists in the concepts of roadworthiness and the legislations between countries on the elements that we use [22], so we also include the definitions of the elements that we consider in the audit to standardize the terms used and avoid confusion at the time of the application of the proposed work.

One of the strengths of our work is its evaluation phase. The preliminary application of the instrument allowed us to observe the limitations that the first version had, in terms of variables and elements considered in the streets; these changes allow a more complete study of the roadway and lead us to an approach to the potential risks faced by drivers and pedestrians who travel along these avenues.

The development of the questionnaire itself carried with it an inherent quantitative methodology, which ensures objectivity and replicability in other contexts where it is applied and can also be implemented for the identification of associated and risk factors. This makes this proposal ideal for the rapid characterization of security problems and the identification of areas of opportunity.

In sum, this work represents a contribution to the field of road safety by developing a road safety inspection constructed through a recognized method and by providing feedback from evaluations of the real context. The individualization of the elements allows a more detailed analysis of the risks and areas of opportunity for the creation of road safety prevention programs.

## 4 Limitations of the study

The authors acknowledge the diversity in roadworthiness concepts and legislation across countries, addressing this by including definitions to standardize terms. The study's strengths lie in its quantitative methodology, ensuring objectivity and replicability, making it applicable in various contexts for identifying associated risk factors and areas of opportunity.

There are other alternatives for evaluating road safety that could complement the one proposed here, such as the incorporation of aspects related to citizen participation through surveys or interviews with the people who frequent the area, where their perception of mobility and safety on the road can be integrated. Also, the use of technologies such as GPS tracking devices or cameras could provide more detailed information on the behavior of road users [[23], [24], [25]].

While the development of the questionnaire involved a quantitative methodology, this approach may not fully capture the nuanced and subjective experiences of road users. The objectivity and replicability claimed could overlook important contextual factors and individual perspectives that contribute to real-world road safety challenges. Additionally, solely relying on quantitative data for the rapid characterization of security problems and identifying areas of opportunity may limit the depth of understanding needed to develop comprehensive and effective solutions. A more balanced approach incorporating both quantitative and qualitative methods could provide a richer and more holistic assessment of road safety conditions [26,27].

## CRediT authorship contribution statement

**Melva Guadalupe Herrera Godina:** Supervision, Formal analysis, Conceptualization. **Guillermo González Estevez:** Resources, Project administration, Investigation, Conceptualization. **Gilberto Silva Bañuelos:** Writing – original draft, Validation, Software, Methodology. **Luis Eduardo del Moral Trinidad:** Writing – review & editing, Writing – original draft, Validation, Methodology, Investigation, Formal analysis.

## Declaration of competing interest

The authors declare that they have no competing interests in relation to the research, design, or implementation of the proposed pedestrian-focused road audit charter. There are no financial, personal, or professional conflicts that could influence or compromise the objectivity and integrity of this study. No funding was given for this article.
