# Supplementary material for: Comprehensive proposal for a pedestrian-focused road safety inspection
Source: Heliyon. 2025 Jan 21;11(3):e42021. doi: 10.1016/j.heliyon.2025.e42021 (PMC11830319; doi:10.1016/j.heliyon.2025.e42021)
Supplement: Multimedia component 1 [file mmc1.docx]

**Annexes**

Annex 1. Risk factors extracted from the literature review.

| Annex I: Risk factors extracted from the review of the literature. | |
| --- | --- |
| **Variable** | **Authors** |
| **Vehicle traffic** | - Abdel-Aty, M., Chundi, S. S., & Lee, C. (2007)(35) - Berhanu, G. (2004)(36) - Donroe, J., Tincopa, M., Gilman, R. H., Brugge, D., & Moore, D. A. (2008)(37) - Hess, P., Moudon, A., & Matlick, J. (2004)(38) - LaScala, E., Gerber, D., & Gruenewald, P. (2000) (1) - LaScala, E., Gruenewald, P., & Johnson, F. (2004) (39) - McMahon, P. J., Duncan, C., Stewart, J. R., Zegeer, C., & Khattak, A. (1999) (40) - Mueller, B. A., Rivara, F. P., Lii, S., & Weiss, N. (1990) (41) - Pulugurtha, S. S., & Sambhara, V. (2011) (42) - Roberts, I., Marshall, R., & Norton, R. (1992) (43) - Roberts, I., & Crombie, I. (1995) (44) - Wier, J., Weintraub, E. H., Seto, E., & Bhatia, R. (2009). (45) |
| **Street length** | - Shuurman, N., Cinnamon, J., Crooks, V. A., & Hameed, S. M. (2009). (46) |
| **Bus stop** | - Clifton, K. J., Burnier, C. V., & Akar, G., (2009) (47) - Hess, P., Moudon, A., & Matlick, J. (2004) (38) - Pulugurtha, S. S., & Sambhara, V. (2011) (42) - Schneider, R. J., Ryznar, R. M., & Khattak, A. J. (2004) (48) - Shuurman, N., Cinnamon, J., Crooks, V. A., & Hameed, S. M. (2009). (46) |
| **Parked car index** | - Agran, P. F., & Winn, D. G. (1996) (49) - Roberts, I., Norton, R., Jackson, R., Dunn, R., & Hassall (1995) (43) - Shuurman, N., Cinnamon, J., Crooks, V. A., & Hameed, S. M. (2009) (46) |
| **Crosswalk** | - Gårder, P. (2004) (50) - Koepsell, T., McCloskey, L., Wolf, M., Muondon, A., Kraus, J., & Patterson, M. (2002) (51) - Mueller, B. A., Rivara, F. P., Lii, S., & Weiss, N. (1990) (41) - Schneider, R. J., Ryznar, R. M., & Khattak, A. J. (2004) (48) - Shuurman, N., Cinnamon, J., Crooks, V. A., & Hameed, S. M. (2009) (46) - Von Kries, R., Kohne, C., Böhm, O., & von Voss, H. (1998) (52) |
| **Number of lanes** | - Abdel-Aty, M., Chundi, S. S., & Lee, C. (2007) (35) - Donroe, J., Tincopa, M., Gilman, R. H., Brugge, D., & Moore, D. A. (2008) (37) - Gårder, P. (2004) (50) - Hess, P., Moudon, A., & Matlick, J. (2004) (38) - Mueller, B. A., Rivara, F. P., Lii, S., & Weiss, N. (1990) (41) - Schneider, R. J., Ryznar, R. M., & Khattak, A. J. (2004) (48) - Shuurman, N., Cinnamon, J., Crooks, V. A., & Hameed, S. M. (2009) (46) |
| **Pedestrian island** | - Constant, A., & Lagarde, E. (2010) (53) - Shuurman, N., Cinnamon, J., Crooks, V. A., & Hameed, S. M. (2009) (46)   • |
| **Signposting** | - Eluru, N., Bhat, C. R., & Hensher, D. A. (2008) (54) - Kim, J., Ulfarsson, G. F., Shankar, V. N. & Mannering, F. L. (2010) - Koepsell, T., McCloskey, L., Wolf, M., Muondon, A., Kraus, J., & Patterson, M. (2002) (51) |
| **Speed limit** | - Abdel-Aty, M., Chundi, S. S., & Lee, C. (2007) (35) - Donroe, J., Tincopa, M., Gilman, R. H., Brugge, D., & Moore, D. A. (2008) (37) - Eluru, N., Bhat, C. R., & Hensher, D. A. (2008).(54) - Gårder, P. (2004) (50) - McMahon, P. J., Duncan, C., Stewart, J. R., Zegeer, C., & Khattak, A. (1999) (40) - Mueller, B. A., Rivara, F. P., Lii, S., & Weiss, N. (1990) (41) - Roberts, I., Norton, R., Jackson, R., Dunn, R., & Hassall (1995) (55) - von Kries, R., Kohne, C., Böhm, O., & von Voss, H. (1998) |
| **Sidewalk** | - Berhanu, G. (2004) (36) - Constant, A., & Lagarde, E. (2010) (53) - McMahon, P. J., Duncan, C., Stewart, J. R., Zegeer, C., & Khattak, A. (1999) (40) - Mueller, B. A., Rivara, F. P., Lii, S., & Weiss, N. (1990) (41) |
| **Road bump** | - Tester, J. M., Rutherford, G. W., Wald, Z., & Rutherford, M. W. (2004) (56) |
| **Street vendors** | - Donroe, J., Tincopa, M., Gilman, R. H., Brugge, D., & Moore, D. A. (2008) (37) |
| **Lane width** | - Agran, P. F., & Winn, D. G. (1996) (49) |
| **Traffic light** | - King, M., Soole, D., & Ghafourian, A. (2009) (57) |
| **Number of pedestrians** | - Agran, P. F., & Winn, D. G. (1996) (49) - Pulugurtha, S. S., & Sambhara, V. (2011) (42) |
| **Type of road** | - Graham, D. J., & Glaister, S. (2003) (58) - Hobday, M. B., & Knigt, S. (2010) (59) - Kim, J., Ulfarsson, G. F., Shankar, V. N. & Mannering, F. L. (2010) (60) - Rothman, L., Slater, M., Meaney, C., & Howard, A. (2010) (61) |
| **Track width** | - Ben-Joseph, E. (1995) (62) - Graham, D. J., & Glaister, S. (2003) (58) |
| **Roadway directions** | - Wazana, A., Rynard, V. L., Raina, P., Krueger, P., & Chambers, L. W. (2000) (63) |
| **Traffic circle** | - Martin, A. J., Hand, E. B., Trece, F., & O’Neill, D. (2010) (64) |

Annex II: Definition of the variables considered in the data collection questionnaire.

| **Variable** | **Definition** |
| --- | --- |
| **Vehicular flow** | Number of vehicles transiting in a given space during 5 minutes |
| **Pedestrian flow** | Number of pedestrians passing through a given space in a given 5-minute period |
| **footbridge** | Road infrastructure work that allows safe pedestrian crossing on roads where vehicular speeds do not permit safe crossing at the level of the road. |
| **Pedestrian island** | Área for pedestrian service and safety |
| **Road bump** | An artificial curb built from curb to curb in the roadway stream to cause vehicle drivers to slow down.  to reduce their speed. |
| **Traffic light** | Electrical or mechanical device that regulates the flow of vehicles and pedestrians at road intersections. |
| **Bus stop** | Place where public transport vehicles stop to pick up or drop off passengers. |
| **Signposting** | Horizontal and vertical road signs, including nomenclature plates, for the orientation of vehicular and pedestrian traffic. |
| **street** | It consists of stream(s) for circulation |
| **Road** | A designated thoroughfare or passageway intended for the movement of motorized vehicles and pedestrians. |
| **Minor- collector** | A road that collects traffic from local streets and channels it into major thoroughfares. |
| **Sub-collector** | A smaller road that connects local streets to minor collectors. Traffic calming devices: Measures used to reduce vehicle speeds and enhance traffic safety. |
| **Tranquilizers** | Measures used to reduce vehicle speeds and enhance traffic safety. |
| **Road way** | The portion of the road dedicated to vehicle travel. |
| **Road width** | The total distance across the road from one edge to the other. |
| **Contraflow lane** | A lane designated for traffic moving in the opposite direction of the normal flow. |
| **Pothole** | A depression or cavity in the road surface. |
| **Traffic circle** | A circular intersection where vehicles circulate around a central island. |
| **Lane demarcation** | Painted lines on the road that define vehicle travel lanes. |
| **Directional arrows** | Arrows painted on the road to indicate the direction of travel. |
| **Traffic lights** | Devices that control the movement of vehicles and pedestrians at intersections. |
| **Speed reducers** | Physical elements designed to slow down traffic. |
| **Crosswalk** | A designated area for pedestrians to safely cross the road. |
| **Pedestrian Island** | A raised area in the road that provides a safe refuge for pedestrians. |
| **Railings** | Barriers placed along the road to protect pedestrians. |
| **Strollers** | Devices used to transport young children, often pushed by pedestrians. |

Annex III. Data collection questionnaire format with the modifications made after field testing.

| I.-Section of the characteristics of the geometric design of the roadway. | | | | | |  |
| --- | --- | --- | --- | --- | --- | --- |
| 1.-Design standards according to the roadway | | | |  |  |  |
| 1.1Type of road __________ | | |  |  |  |  |
|  | 1) Controlled access | |  |  |  |  |
|  | 2) Main |  |  |  |  |  |
|  | 3) Collector |  |  |  |  |  |
|  | 4) Minor collector | |  |  |  |  |
|  | 5) Sub-collector | |  |  |  |  |
|  | 6) Local |  |  |  |  |  |
|  | 7) Tranquilizers | |  |  |  |  |
|  |  |  |  |  |  |  |
| 1.2.-How many ways is the road? | | |  | 1 __________ 2 __________ | | |
|  |  |  |  |  |  |  |
| 1.3.-If it is a two-way road, does it have physical separation in each direction? | | | | | |  |
|  | Yes __________ Width __________ m No __________ | | | | |  |
| 1.4.-What is the width of the road? | | |  |  |  |  |
|  |  |  |  |  |  |  |
|  |  |  |  |  |  |  |
|  |  |  |  |  |  |  |
| 1.5.-Is there a contraflow lane present? Yes __________ No __________ | | | | | |  |
|  |  |  |  |  |  |  |
| 1.6.-Is the street : | | __________ |  |  |  |  |
|  | 1) Asphalt |  |  |  |  |  |
|  | 2) Cobblestone | |  |  |  |  |
|  | 3) Concrete |  |  |  |  |  |
|  | 4) Cobblestone | |  |  |  |  |
|  | 5) Terracing |  |  |  |  |  |
|  |  |  |  |  |  |  |
| 1.7.-The street has a curb and gutter: Yes __________ No __________ | | | | | |  |
|  |  |  |  |  |  |  |
| 1.7.1.-How high is it? | | __________ m | |  |  |  |
|  |  |  |  |  |  |  |
| 1.8.-What type of intersection is it? | | | __________ |  |  |  |
|  |  |  |  |  |  |  |
| 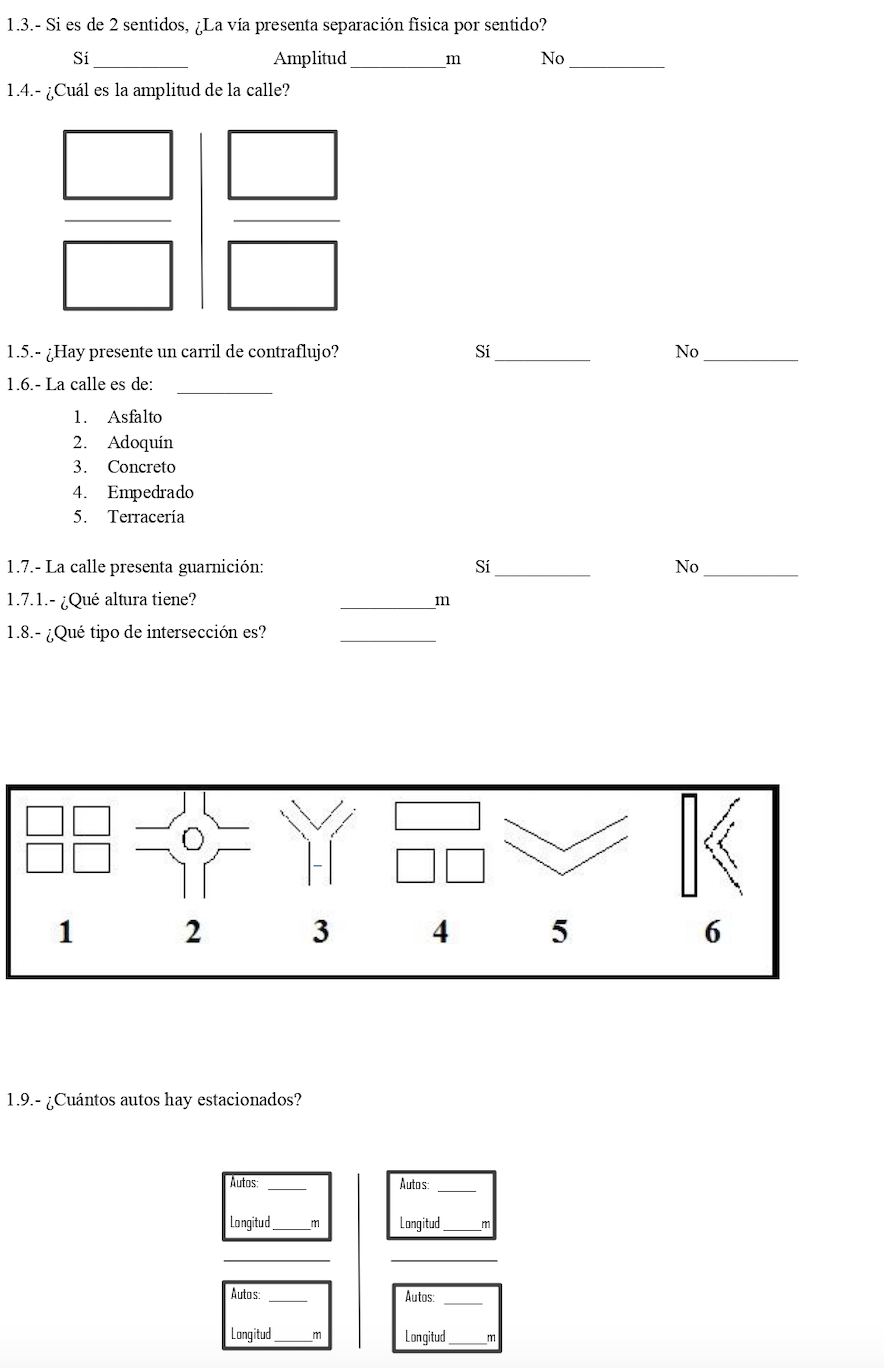   \|  \| \| --- \| |  |  |  |  |  |  |
|  |  |  |  |  |  |  |
|  |  |  |  |  |  |  |
|  |  |  |  |  |  |  |
|  |  |  |  |  |  |  |
|  |  |  |  |  |  |  |
|  |  |  |  |  |  |  |
|  |  |  |  |  |  |  |
| 1.9.-How many cars are there parked? | | |  |  |  |  |
|  |  |  |  |  |  |  |
|  | Cars __________ Cars __________ | | |  |  |  |
|  | Length ________m Length ________m | | | |  |  |
|  | Cars Cars __________ | |  |  |  |  |
|  | Length ________m Length ________m | | | |  |  |
|  |  |  |  |  |  |  |
| 1.10.-Height 1 __________ masl Height 2 __________ masl Distance __________ | | | | | | |
|  |  |  |  |  |  |  |
| 1.11.-Does the road have potholes? | | |  | Yes __________ No __________ | | |
|  |  |  |  |  |  |  |
| II.Section of the traffic circle characteristics | | | |  |  |  |
| 2.1.-Is there a traffic circle in the area? | | | Yes __________ No __________ | | |  |
| (If you answer no to this question go to section III) | | | |  |  |  |
|  |  |  |  |  |  |  |
| 2.2.-Is the traffic circle signposted? | | | Yes __________ No __________ | | |  |
|  |  |  |  |  |  |  |
| 2.3.-What is the diameter of the traffic circle? | | | | __________ m | |  |
|  |  |  |  |  |  |  |
| III.-Signaling section | |  |  |  |  |  |
| 3.1.-Vertical Signals | |  |  |  |  |  |
| 3.1.1.-Signals are present at the site: | | |  |  |  |  |
| Stop Yes __________ No __________ Visibility B __________ M __________ | | | | | |  |
| Yield Yes __________ No __________ Visibility B __________ M __________ | | | | | |  |
| Speed Limit Yes __________ No __________ Visibility B __________ M __________ | | | | | | |
| (If you answer yes to this section write down what is the speed limit __________Km/h) | | | | | | |
|  |  |  |  |  |  |  |
| 3.2.-Horizontal Signs | |  |  |  |  |  |
| 3.2.1.-Signs are present in the place: | | |  |  |  |  |
| Stop Yes __________ No __________ Status B __________ M __________ | | | | | |  |
| Lane demarcation Yes __________ No __________ Status B __________ M __________ | | | | | | |
| Directional arrows Yes __________ No __________ State B __________ M __________ | | | | | | |
| Speed limit Yes __________ No __________ Status B __________ M __________ | | | | | | |
| (If you answer yes to this section write down what is the speed limit __________Km/h) | | | | | | |
|  |  |  |  |  |  |  |
| 3.2.2.-Are there any previous visible demarcations? | | | | Yes __________ No __________ | | |
|  |  |  |  |  |  |  |
| IV.-Section of the bus stop characteristics | | | |  |  |  |
| 4.1.-There is a bus stop on the road Yes __________ No __________ | | | | | |  |
| (If there is no bus stop go to section V) | | |  |  |  |  |
|  |  |  |  |  |  |  |
|  | Official __________ Non-official __________ | | | |  |  |
|  |  |  |  |  |  |  |
| 4.2.-Is it vertically signposted? | | | Yes __________ No __________ | | |  |
| 4.3.-Is it marked? | |  | Yes __________ No __________ | | |  |
| 4.4.-How wide is the traffic island? | | | __________ m | |  |  |
| 4.5.-Are there cars parked in the bus stop space? | | | |  |  | Yes __________ No __________ |
| 4.6.-Where is the bus stop located? | | |  |  |  |  |
| Before __________ After __________ of the intersection | | | | |  |  |
| Before __________ After __________ of the crosswalk | | | | |  |  |
|  |  |  |  |  |  |  |
| V.-Section of traffic light characteristics | | | |  |  |  |
| 5.1.-Are there traffic lights in the area? | | | | Yes __________ No __________ | | |
| (If there is no traffic light go to section VI) | | | |  |  |  |
| 5.2.-Do they have an exclusive time for pedestrians? | | | | Yes __________ No __________ | | |
| 5.3.-How long does it last? | | __________ s | |  |  |  |
| 5.4.-Is there a traffic light for pedestrians? | | | | Yes __________ No __________ | | |
| 5.5.-How long does it last? | | __________ s | |  |  |  |
| 5.6.-Do the traffic lights have good visibility? | | | | Yes __________ No __________ | | |
| 5.7.-Is it possible to be dazzled by the sun? | | | |  | Yes __________ No __________ | |
|  |  |  |  |  |  |  |
| VI.-Speed reducers section | | |  |  |  |  |
| 6.1.-Are any of the following speed reducers present? | | | | |  |  |
| Lane number decrease Yes __________ No __________ | | | | |  |  |
| Stop Yes __________ No __________ | | |  |  |  |  |
|  |  |  |  |  |  |  |
| VII.-Section of the pedestrian subsystem characteristics | | | | |  |  |
| 7.1.-Pedestrian walkway | |  |  |  |  |  |
| 7.1.1.-Is there a marked crosswalk? | | |  |  |  |  |
| 7.1.2.-Is it in good condition? | | |  |  |  |  |
| 7.1.3.-Does it have a different texture than the rest of the street? | | | | |  |  |
|  |  |  |  |  |  |  |
| 7.2.-Is there a pedestrian island in the area? | | | | Yes __________ No __________ | | |
| 7.2.1.-How long is it? | |  | __________ m | |  |  |
| 7.2.2.-Does it have railings? | | |  | Yes __________ No __________ | | |
| 7.2.3.-Is it marked? | |  |  | Yes __________ No __________ | | |
| 7.2.4.-Does it have space for strollers? | | |  | Yes __________ No __________ | | |
|  |  |  |  |  |  |  |
| Is there a pedestrian bridge in the area? | | | | Yes __________ No __________ | | |
| 7.3.1.-Is it signposted? | |  |  | Yes __________ No __________ | | |
| 7.3.2.-Is it illuminated? | |  |  | Yes __________ No __________ | | |
| 7.3.3.-Entrance by: | |  |  | Stairs __________ Ramp __________ | | |
|  |  |  |  |  |  |  |
| 7.4.-Are there sidewalks in the area? | | |  | Yes __________ No __________ | | |
| 7.4.1.-Is the area wheelchair accessible? | | | | Yes __________ No __________ | | |
| 7.4.2.-How wide is it? | |  | __________ m | |  |  |
| 7.4.3.-Are there any of the following elements invading the sidewalk? | | | | | |  |
|  | Cars Yes __________ No __________ | | |  |  |  |
|  | Street stores Yes __________ No __________ | | | |  |  |
|  |  |  |  |  |  |  |
| 7.4.4.-The sidewalk presents: | | |  |  |  |  |
|  | Continuity Yes __________ No __________ | | | |  |  |
|  | Slopes Yes __________ No __________ | | | |  |  |
|  | Steps Yes __________ No __________ | | |  |  |  |
